# Dental Implant Surrounding Marginal Bone Level Evaluation: Platform Switching versus Platform Matching—One-Year Retrospective Study

**DOI:** 10.1155/2017/7191534

**Published:** 2017-10-24

**Authors:** Eisner Salamanca, Jerry C.-Y. Lin, Chi-Yang Tsai, Yung-Szu Hsu, Haw-Ming Huang, Nai-Chia Teng, Peter D. Wang, Sheng-Wei Feng, May-Show Chen, Wei-Jen Chang

**Affiliations:** ^1^School of Dentistry, College of Oral Medicine, Taipei Medical University, Taipei 110, Taiwan; ^2^Department of Oral Medicine, Infection and Immunity, Harvard School of Dental Medicine, Boston, MA, USA; ^3^Dental Department, Taipei Medical University Hospital, Taipei 110, Taiwan; ^4^School of Oral Hygiene, College of Oral Medicine, Taipei Medical University, Taipei 110, Taiwan; ^5^Dental Department, Taipei Medical University, Shuang-Ho Hospital, Taipei 235, Taiwan

## Abstract

The benefits and feasibility of platform switching have been discussed in several studies, reporting lesser crestal bone loss in platform-switched implants than in platform-matched implants.* Objective.* The aim of the present study was to observe the changes in vertical and horizontal marginal bone levels in platform-switched and platform-matched dental implants.* Materials and Methods.* 51 patients received 60 dental implants in the present study over a 1-year period. Measurement was performed between the implant shoulder and the most apical and horizontal marginal defect by periapical radiographs to examine the changes of peri-implant alveolar bone before and 12 months after prosthodontic restoration delivery.* Results*. These marginal bone measurements showed a bone gain of 0.23 ± 0.58 mm in the vertical gap and 0.22 ± 0.53 mm in the horizontal gap of platform matching, while in platform switching a bone gain of 0.93 ± 1 mm (*P* < 0.05) in the vertical gap and 0.50 ± 0.56 mm in the horizontal gap was found. The average vertical gap reduction from the baseline until 12 months was 0.92 ± 1.11 mm in platform switching and 0.29 ± 0.85 mm in platform matching (*P* < 0.05).* Conclusions.* Within the limitations of the present study, platform switching seemed to be more effective for a better peri-implant alveolar bone vertical and horizontal gap reduction at 1 year.

## 1. Introduction

Dental implant therapy is considered an appropriate treatment for edentulous and partially dentulous patients since Dr. Brånemark began performing dental implant operations on edentulous patients in 1965. In addition, it has become the ideal method of oral rehabilitation after missing natural dentition and has been recognized as a reliable and predictable tool for dental reconstruction, necessitating that multiple factors are reached for long-term treatment success and esthetics.

Evaluation of circumferential bone loss around dental implants by using periapical radiographs has been frequently used in routine clinical practice to prevent treatment failure and ensure favorable long-term prognosis. This method of evaluation has been debated, where certain authors have reported bone reabsorption rates around dental implants: for example, Adell et al. [1] reported that radiographic crestal bone loss during the first year after abutment connection was 1.2 mm, with a mean vertical bone loss of <0.2 mm annually following function, in both mandible and maxilla. Subsequently, Albrektsson proposed that a dental implant can be considered successful if the peri-implant crestal bone loss in the first year is <1.5 mm, and the ongoing annual bone loss is <0.2 mm [2]. Moreover, findings from several other studies have indicated that the long-term results of endosseous implants primarily depend on preservation of bone support [3–7]. Therefore, the maintenance of osseointegration and a stable marginal bone level is necessary for the success of a dental implant.

Gardner, Porter, and Lazzara introduced the concept of platform switching, by which a larger-diameter implant is combined with a narrower abutment, resulting in movement of the implant-abutment gap away from the implant shoulder. The benefits and feasibility of platform switching have been discussed in several studies, reporting lesser crestal bone loss in platform-switched implants than in platform-matched implants [8–14]. Some of the reasons for the success of platform-switched implants in terms of minimum crestal bone height changes include implant loading and concentration of forces, the countersinking procedure during implant placement, and localized soft tissue inflammation [13].

The aim of the present study was to observe the changes in both vertical and horizontal marginal bone defect measured between the implant shoulder and the most apical and horizontal marginal defect by using periapical radiographs to examine the changes in mesial and distal peri-implant alveolar bone before and 12 months after prosthodontic restoration delivery between platform-switched and platform-matched dental implants.

## 2. Materials and Methods

### 2.1. Patient Selection

Patients requiring single-tooth extraction were selected according to the following selection criteria: good systemic health; nonsmoking or smoking ≤ 10 cigarettes/day; good oral hygiene; full-mouth plaque score (FMPS) ≤ 25% at baseline; full-mouth bleeding of probing (FMBS) ≤ 25% at baseline; probing pocket depth (PPD) at six aspects of the teeth adjacent to the implant site ≤ 3 mm; periodontal attachment level (PAL) at six aspects of the teeth adjacent to the implant site ≤ 2 mm; absence of active infection around the surgical site; presence of natural teeth adjacent to the implant site; adequate bone tissue to ensure implant primary stability; presence of keratinized tissue (KT) ≥ 2 mm; stable posterior occlusion; absence of parafunctional habits (bruxism, clenching).

The exclusion criteria were as follows: patients with any local or systemic disease, smoking more than 10 cigarettes/day, betel nut or tobacco chewing, alcoholism, pregnancy or breastfeeding, long-term oral medications, oral parafunction, nontreated periodontal disease; inadequate bone volume; inability to maintain obligation to implant treatment and maintenance; and inability or reluctance to provide informed consent.

### 2.2. Surgical Procedure

This retrospective clinical study was performed in compliance with good clinical practice and the principles outlined in the Declaration of Helsinki, last revised in Edinburgh in 2000. All protocols were registered and approved by Taipei Medical University Joint Institutional Review Board (approval number 201301034). The study patients were aged between 28 and 80 years. Dentium implants were early placed 6 weeks after tooth extraction. Patients received local anesthesia, a midcrestal incision was performed in the edentulous area, and full-thickness mucoperiosteal flap was elevated. Later, following the manufacturer's surgical protocol, dental implants were placed and flaps were sutured. Subjects received postoperative instructions and were advised to rinse with chlorhexidine 0.12% twice a day for 10 days, and sutures were removed 2 weeks after. All implants were inserted until the outer edge of the dental implant reached the marginal bone level, to allow for the apex of the cover screw to be at level with the bone crest during the healing period [13]. Surgical and prosthetic restoration procedures were performed by the same trained dental surgeon in a total of 51 patients between March 2010 and January 2015 at the Shuang-Ho Hospital, New Taipei, Taiwan. At 3 months after implant insertion, a secondary surgery following the first surgery protocol was carried out for healing abutments insertion, and the end flaps were sutured around the connected healing abutments. Patients followed the same postsurgical instructions as the ones given in the first surgery.

The criteria established by Buser et al. (1990) [15] were used to determine the implants success by defining the following: whether the implant is in situ; no persistent complaints such as pain, discomfort, or paresthesia; no peri-implant infection with suppuration; no mobility of the implant; no peri-implant radiolucency. Additionally, implant success was estimated combining the criteria established by Buser with the criterion of bone loss < 1 mm (mean of mesial and distal measurements).

### 2.3. Prosthetic Procedure

Implant level impressions were taken 6 weeks postoperatively to the healing abutment surgery connection. The permanent metal ceramic crown was delivered 2 weeks after impressions. Overall, 27 patients received 30 platform-matched dental implants (diameter: 3.4–4.5 mm; length: 9.5–13 mm); on the other hand, 24 patients received 30 platform-switched dental implants (diameter: 3.3–4.8 mm; length: 8–12 mm), with a horizontal circular step of 0.6 mm between the outer edge of the dental implant and the narrower abutment. Both groups were followed up for 12 months after the final prosthetic restoration was delivered. A maximum of 3 implants were placed in one patient, and each implant was placed in a different quadrant to replace one missing tooth. Each implant measured at least 3.3 mm in width and at least 8 mm in length.

### 2.4. Radiographic Examination

**Figure 1:**
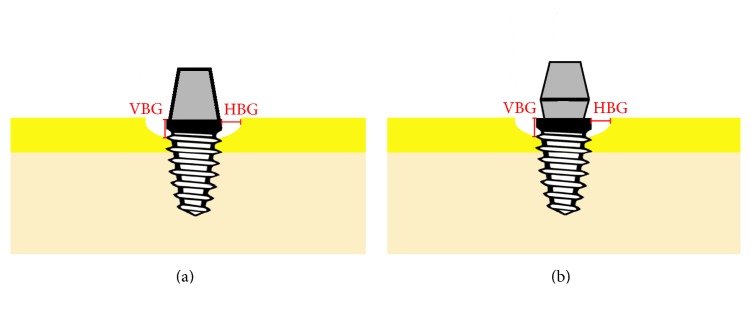
Radiographic measurements of dental implants. (a) Platform-matched dental implants. (b) Platform-switched dental implants. VBG: vertical bone gap; HBG: horizontal bone gap.

Standardized digital intraoral radiographs were recorded by following the long-cone paralleling technique, as described by Meijndert et al. [16]. Digital periapical radiographs of the dental implants were recorded at different time points: before loading (baseline); immediately after loading; and 1, 3, 6, and 12 months after loading. The implant shoulder was considered as the reference point for measuring vertical and horizontal dimensions (vertical bone gap and horizontal bone gap) of the mesial and distal peri-implant marginal bone defect; the same measurements were used to evaluate bone remodeling through the 12 months of follow-up. EZ-Dental professional imaging software (Asahi Co. Ltd., Tokyo, Japan) was used for measurements. Results were recorded and collected by two experienced examiners, different from the dental surgeon who performed the implant placement (Figure 1). The length calibration tool of the EZ-Dental professional imaging software was used to correct the deviation of the periapical films. Calibration of periapical films was fulfilled by inputting the real length of the dental implant. Thereafter, the length-measuring tool was used to obtain the mesial and distal measurements of vertical and horizontal bone gaps after calibration and at 1, 3, 6, and 12 months after loading.

### 2.5. Statistical Analysis

Descriptive statistics including mean values and standard deviations were used. For testing normality, Jarque-Bera test was used. The analysis was based on “dental implant” as the unit. Independent and paired sample* t-*tests were conducted and comparisons were computed by means with repeated measures within and between groups, respectively. Statistical significance was set at *P* < 0.05 for all statistical tests. All tests were conducted at the 95% level of confidence. The statistical evaluation of the difference in mesial and distal marginal bone gap loss was accomplished with independent* t-*test. Microsoft Excel Professional Plus 2016 (Microsoft Software, Redmond, WA) was used for all data analyses.

## 3. Results

No significant differences in demographic data were found between the groups. In total, 51 patients (30 men and 21 women) received 60 dental implants in the present study. Overall, 30 platform-matched implants were implanted in a total of 27 patients (19 men, mean age: 53.7 ± 19.7 years; 8 women, mean age: 54.2 ± 26 years). On the other hand, 30 platform-switched implants were placed in 24 patients (11 men, mean age: 53.1 ± 28 years; 13 women, mean age: 54.3 ± 15.7 years). Tables 1 and 2 detail vertical and horizontal bone gap results in platform-switched and platform-matched implant groups.

**Figure 2:**
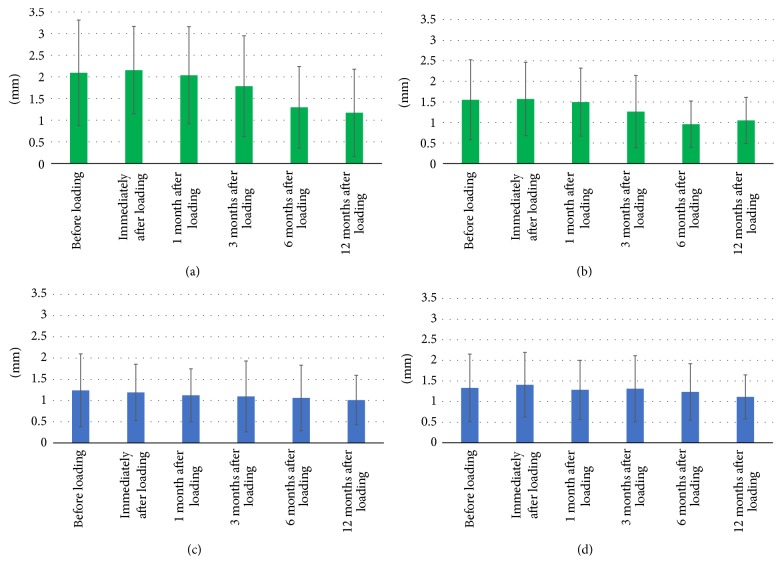
Vertical and horizontal gap measurements over 12-month follow-up. Vertical and horizontal mean marginal bone level variations before and up to 12 months after loading. (a) Platform-switching average vertical gap measurements during 12 months. (b) Platform-switching average horizontal gap measurements during 12 months. (c) Platform matching average vertical gap measurements during 12 months. (d) Platform matching average horizontal gap measurements during 12 months.

In Table 1, the vertical bone gap variations from platform-switched implants appear. The mean vertical bone gap in platform-switched implants was 2.09 ± 1.22 mm mean before loading; 2.15 ± 1.01 mm mean immediately after loading; 2.04 ± 1.12 mm mean 1 month after loading; 1.79 ± 1.16 mm mean 3 months after loading; 1.3 ± 0.94 mm mean 6 months after loading; and 1.17 ± 1.01 mm mean 12 months after loading. Statistical analysis showed a statistically significant difference (*P* < 0.05) between the baseline and 6 months and between the baseline and 12 months in all the vertical measurements (Figure 2(a), Table 1).

Horizontal bone gap variations in platform-switched implants appear in Table 1. The mean horizontal bone gap in platform-switched implants was 1.55 ± 0.97 mm mean before loading; 1.57 ± 0.89 mm mean immediately after loading; 1.49 ± 0.83 mm mean 1 month after loading; 1.26 ± 0.88 mm mean 3 months after loading; 0.96 ± 0.56 mm mean 6 months after loading; and 1.05 ± 0.56 mm mean 12 months after loading. Statistical analysis showed a statistically significant difference (*P* < 0.05) between the baseline and 6 months and between the baseline and 12 months in all the horizontal measurements (Figure 2(b), Table 1).

Table 2 shows the vertical marginal bone gap variations in platform-matched implants during the 12-month study period. The mean vertical bone gap in platform-matched implants was 1.24 ± 0.86 mm mean before loading; 1.19 ± 0.66 mm mean immediately after loading; 1.12 ± 0.63 mm mean 1 month after loading; 1.1 ± 0.83 mm mean 3 months after loading; 1.06 ± 0.77 mm mean 6 months after loading; and 1.01 ± 0.58 mm mean 12 months after loading. Statistical analysis showed only a significant difference (*P* < 0.05) between the baseline and 12 months in distal measurements (Figure 2(c), Table 2).

The horizontal bone gap results in platform-matched implants are displayed in Table 2. The mean horizontal bone gap in platform-matched implants was 1.33 ± 0.82 mm mean before loading; 1.41 ± 0.78 mm mean immediately after loading; 1.29 ± 0.72 mm mean 1 month after loading; 1.31 ± 0.8 mm mean 3 months after loading; 1.23 ± 0.68 mm mean 6 months after loading; and 1.11 ± 0.53 mm mean 12 months after loading. Statistical analysis showed no statistically significant differences between the baseline and the rest of the time points in any of the horizontal measurements (Figure 2(d), Table 2).

**Figure 3:**
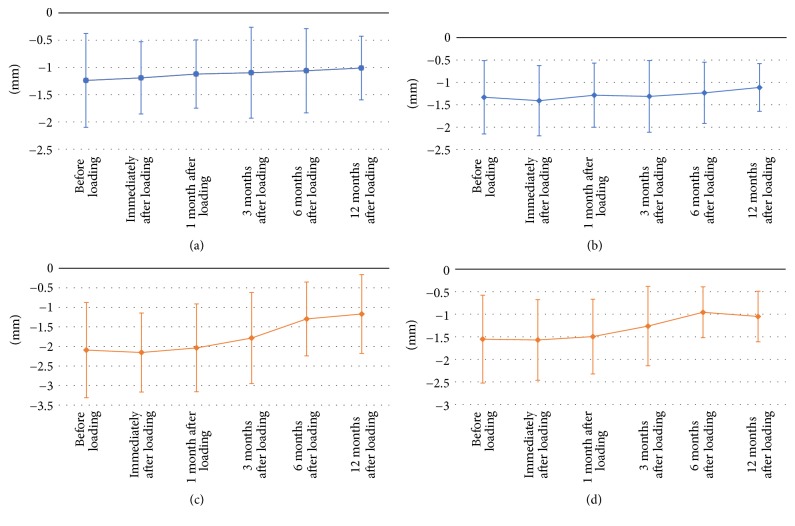
Vertical and horizontal bone gain over 12-month follow-up. Vertical and horizontal bone gain in marginal bone defects before and up to 12 months after loading. (a) Platform matching vertical bone variations average measurements. (b) Platform matching horizontal bone variations average measurements. (c) Platform-switching vertical bone variations average measurements. (d) Platform-switching horizontal bone variations average measurements.

These marginal bone measurements showed a bone gain of 0.23 ± 0.58 mm in the vertical gap and 0.22 ± 0.53 mm in the horizontal gap of the platform matching, while in the platform switching a bone gain of 0.93 ± 1 mm in the vertical gap (*P* < 0.05) and 0.50 ± 0.56 mm in the horizontal gap was found. Only a statistically significant difference was found comparing bone gains in the vertical gap between the two groups (*P* < 0.05) (Figure 3).

**Figure 4:**
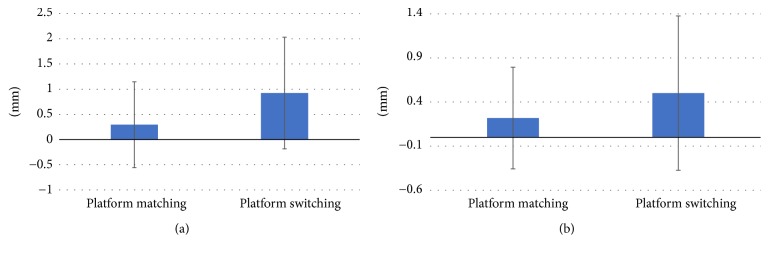
Platform matching versus platform-switching average vertical and horizontal gap reduction during 12 months. Vertical and horizontal gap reduction during 12 months. (a) Average vertical gap reduction at 12 months. (b) Average horizontal gap reduction at 12 months.

The average vertical gap reduction from the baseline until 12 months was 0.92 ± 1.11 mm in platform switching and 0.29 ± 0.85 mm in platform matching (*P* < 0.05). The average horizontal gap reduction from the baseline until 12 months was 0.50 ± 0.87 mm in platform switching and 0.22 ± 0.58 mm in platform matching (Figure 4).

## 4. Discussion

The aim of this study was to evaluate the changes in both vertical and horizontal bone gaps between the peri-implant crestal bone and the dental implant surface. Periapical radiographs were used to examine the peri-implant alveolar bone changes before loading and 12 months after the final prosthodontic restoration was delivered. Clinically, all implants exhibited osseointegration, with a 100% success rate. The short-term survival rate in the present 1-year retrospective study might justify the expectation of successful long-term survival, as there is sufficient evidence that implant losses mainly occur within he first month after placement [17]. Overall, both platform-switched and platform-matched implant groups exhibited reduced vertical and horizontal gaps at the end of the 12 months. On comparison, the mean marginal bone gaps showed more reduction in the platform-switched dental implants, with only statistically significant differences between the two groups at the end of the 12 months in the vertical measurements, where platform-switched implants presented more mean reduction in the vertical marginal bone gap (0.93 ± 1 mm) than the platform-matched implants did (0.29 ± 0.85 mm). Similar results have been reported in previous studies, where marginal bone levels were better maintained in platform-switched implants [18]. In addition, the platform-switching concept helps obtain satisfactory long-term esthetic results by the mean marginal bone reduction obtained in vertical and horizontal gaps [19, 20].

Previous studies have suggested that the position of marginal bone after delivery of the final prosthetic restoration can be determined by several factors that control the crestal bone levels around dental implants: a minimum of 3 mm of soft tissue, which is necessary for the position of the implant-abutment junction where cells infiltrate, and its proximity to the crestal bone and the implant surface topography [13]. In the present study, the soft tissue above the dental implant was not measured because, despite the general suggestion of a minimum of 3 mm of mucosal thickness that allows the establishment of a biological width, Berglundh and Lindhe reported greater crestal bone loss when the soft tissue surrounding an implant was intentionally reduced to ≤2 mm [21]. However, more recently, other authors have found only significant differences in bone loss between switched and matched sides at a mean of 4.22 (1.50–7.00) mm of soft tissue thickness [22]. Thus, in the present study, we only focused on bone tissue changes. Despite having a statistically significant difference observed in the mean reduction in the vertical gap between the two groups at 12 months from platform switching over platform matching, in the horizontal marginal bone defect reduction at 12 months, platform switching had 0.50 ± 0.87 mm reduction while platform matching had 0.22 ± 0.58 mm; the comparison between the two reductions showed no statistically significant difference.

A recent systematic review and meta-analysis study with a total of 26 studies involving 1,511 platform-switched implants and 1,123 platform-matched implants indicated that platform switching within 18 months following crown placement had lower vertical marginal bone loss (0.23 mm) compared to platform-matched implants. After more than 1 year of function, slight soft tissue loss was observed in platform-switched implants; hence, the results of soft tissue should be interpreted with caution for a better long-term successful treatment. The authors concluded that platform switching may have an indirect protective effect on implant hard tissue outcomes [23]. They also pointed out the difficulty in evaluating the impact of thick tissue on marginal bone preservation in conjunction with the platform-switching concept. In accordance with the systematic review and meta-analysis study, the present study also had the same tendency with less vertical bone loss in platform switching than in platform matching.

**Figure 5:**
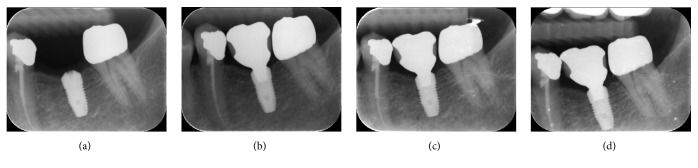
Radiographic variations during 12 months after loading in platform-switched dental implants. (a) Baseline, before loading; (b) immediately after loading; (c) 6 months after loading; (d) 12 months after loading.

The implants used in this study were sandblasted with large-grit and acid-etched surfaces and had been tested in previous studies, demonstrating good bone-to-implant contact and clinical performance by maintaining good crestal bone height like the results obtained in the present study [24]. Considering the dental implant design, the implant-abutment junction was left at the marginal bone level. All implants had the same design regardless of being used in the platform-switched or platform-matched groups. Findings from the present study revealed that although the vertical and horizontal bone gaps were reduced at the end of the 12 months in comparison with the gaps before loading, there was minimal marginal bone remodeling in all cases; this could be attributed to the biological bone remodeling that occurs in the first year after dental implant loading (Figure 5). In concordance with results of a systematic review and meta-analysis study, platform switching for marginal bone preservation around dental implants and an abutment/implant diameter difference of ≥0.4 mm were associated with a more favorable bone response [25]. Despite the short study period of 12 months, minimal crestal bone remodeling with bone gain and marginal bone gap reduction could be noted during the study, which agrees with results reported by Cappiello et al. and other authors who conducted studies over longer time periods, indicating that platform switching seemed to reduce the peri-implant crestal bone resorption and increase the long-term predictability of implant therapy [9, 18, 26].

Platform-switched implant procedure presents several potential disadvantages such as the need for components that have similar designs (the screw access hole must be uniform) and the need for enough space to develop an appropriate emergence profile [19]. This procedure shifts the stress concentration away from the bone-implant interface, but these forces are subsequently increased in the abutment or the abutment screw [27]. Thus, the dental implant used in this study was ideal for this type of approach because of its simple system of single abutment with internal conical connection between the implant and the abutment interface, eliminating any possible disadvantages of platform switching and being stable with platform matching during the study period.

## 5. Conclusion

With the limitations of the present study, platform switching seemed more effective for a better peri-implant alveolar bone vertical and horizontal gap reduction at 1 year. Despite the abutment connection used, the dental implant in the present study presented minimal bone gain at the marginal bone level, indicating a good long-term treatment prognosis. Further studies with longer periods of time and impact of thick tissue on marginal bone preservation in conjunction with the platform-switching concept are needed.

## Figures and Tables

**Table 1:** Platform switching (mean ± SD, mm).

	Vertical			Horizontal		
	Mesial	Distal	Mean	Mesial	Distal	Mean
Baseline	2.06 ± 1.28	2.13 ± 1.29	2.09 ± 1.22	1.97 ± 1.39	1.13 ± 0.83	1.55 ± 0.97
Immediately after loading	2.15 ± 1.04	2.16 ± 1.17	2.15 ± 1.01	1.89 ± 1.32	1.25 ± 0.82	1.57 ± 0.89
1 month after loading	1.96 ± 1.12	2.11 ± 1.25	2.04 ± 1.12	1.97 ± 1.2	1.02 ± 0.79	1.49 ± 0.83
3 months after loading	1.73 ± 1.25	1.84 ± 1.14	1.79 ± 1.16	1.63 ± 1.23	0.9 ± 0.72	1.26 ± 0.88
6 months after loading	1.29 ± 1.02	1.3 ± 0.99	1.3 ± 0.94	1.28 ± 1.01	0.63 ± 0.63	0.96 ± 0.56
12 months after loading	1.02 ± 0.99	1.32 ± 1.17	1.17 ± 1.01	1.27 ± 0.85	0.83 ± 0.5	1.05 ± 0.56

**Table 2:** Platform matching (mean ± SD, mm).

	Vertical			Horizontal		
	Mesial	Distal	Mean	Mesial	Distal	Mean
Baseline	1.14 ± 1	1.33 ± 1.06	1.24 ± 0.86	1.46 ± 1.19	1.2 ± 1.03	1.33 ± 0.82
Immediately after loading	1.16 ± 0.75	1.23 ± 0.76	1.19 ± 0.66	1.63 ± 1.24	1.18 ± 1.02	1.41 ± 0.78
1 month after loading	1.01 ± 0.68	1.23 ± 0.78	1.12 ± 0.63	1.53 ± 1.11	1.04 ± 0.93	1.29 ± 0.72
3 months after loading	1.06 ± 1.02	1.13 ± 0.8	1.1 ± 0.83	1.52 ± 1.24	1.1 ± 0.98	1.31 ± 0.8
6 months after loading	1.01 ± 0.75	1.1 ± 0.9	1.06 ± 0.77	1.51 ± 1.03	0.95 ± 0.79	1.23 ± 0.68
12 months after loading	1.07 ± 0.71	0.96 ± 0.68	1.01 ± 0.58	1.27 ± 0.73	0.95 ± 0.73	1.11 ± 0.53
